# PsWRKY75 orchestrates lignin biosynthesis and H₂O₂ metabolism to modulate disease resistance: a physiological and biochemical mechanism in *Prunus sibirica* L.

**DOI:** 10.1186/s12870-026-09403-y

**Published:** 2026-07-07

**Authors:** Qiuxian Bai, Haiyan Lan, Dan Liu

**Affiliations:** Key Laboratory of Protection, Development and Utilization of Medicinal Resources in Liupanshan Area, Ministry of Education, School of Pharmacy, Ningxia Medical University, Yinchuan, Ningxia Hui Autonomous Region 750004 P. R. China

**Keywords:** *Prunus sibirica* L., Disease resistance, *PsWRKY75*, H_2_O_2_, Lignin accumulation

## Abstract

*Prunus sibirica* L., an economically and ecologically significant Asian endemic species, faces severe pathogenic threats, yet its disease resistance mechanisms remain poorly understood. Using RNA-seq of pathogen-infected leaves, we identified PsWRKY75 as a potential resistance gene. Heterologous overexpression in poplars showed that PsWRKY75 enhanced disease resistance, promoting lignin and H₂O₂ accumulation in leaves. Through RNA-seq and promoter analysis, PsWRKY75 was identified as an upstream regulator of the H₂O₂-producing gene *PsRbohB* and lignin biosynthesis gene *PsLFP1*, validated by yeast one-hybrid, EMSA, and dual luciferase assays. Pathogen infection upregulates PsWRKY75, which directly activates *PsRbohB* and *PsLFP1* to enhance lignin/H₂O₂ accumulation, thereby strengthening disease resistance. This study reveals PsWRKY75 as a novel integrator of H₂O₂ signaling and lignin biosynthesis, providing genetic resources for woody plant disease resistance improvement via physiological and biochemical engineering.

**Supplementary Information:**

The online version contains supplementary material available at https://doi.org/10.1186/s12870-026-09403-y.

## Introduction

*Prunus sibirica* L. is an ecological and economic tree species unique to Asia, widely distributed in eastern Siberia, eastern Mongolia, and the northeastern and northern regions of China [1]. *P. sibirica* L. can grow healthily in areas with low rainfall, low temperatures, and relatively infertile soil. As an ecological species, it plays functions such as water conservation, sand fixation, and greening. Meanwhile, oil can be extracted from seeds for use in edible oil, cosmetics, and medicine [2]. Therefore, *P. sibirica* L. has played a positive role in promoting the regional economic development in China. However, during the production process, *P. sibirica* L. has long been threatened by diseases such as brown rot, bacterial shot hole, and gummosis, which seriously affect the growth and utilization rate of fruits and seeds. Therefore, it is of great theoretical and practical significance to explore the disease resistant mechanism of *P. sibirica* L. and excavate the key regulatory genes. However, how transcription factors coordinate ROS metabolism and lignin biosynthesis at the physiological level remains poorly understood in *Prunus* species. Recently, the analysis of the genome of its related species *Prunus armeniaca* has provided an important theoretical basis for the gene mining and reverse genetics research on the trait regulation of *Prunus* species [3].

Plants have evolved diverse active and passive defense strategies against pathogenic attacks. Passive barriers consist of inherent tissue structures and pre-synthesized metabolites (such as phenolics, unsaturated lactones, and antimicrobial peptides) that block pathogen entry [4]. Active defense responses, by contrast, involve generating antitoxins, reactive oxygen/nitrogen species (ROS/RNS), reinforcing cell walls, and triggering hypersensitive reactions [5]. Together, active and passive defenses determine a plant’s susceptibility to pathogens, where the presence of disease-resistant genes genetically controls whether plants are susceptible to diseases or avirulence determinants [6]. Numerous genes actively participate in plant disease resistance through various mechanisms. For example, the Ca²⁺ receptor proteins CML13 and CML8 exhibit defensive functions against multiple pathogens, including *Pseudomonas syringae* and *Ralstonia solanacearum*. Their overexpression regulates the expression of a series of pathogenesis-related (PR) genes [7, 8]. Another Ca²⁺-dependent protein kinase, CPK5, enhances resistance to *P. syringae* pv. *tomato* strain DC3000 by promoting defense gene expression and ROS synthesis via a salicylic acid-mediated pathway [9]. Transcription factors ERF1 and MYC2 integrate jasmonic acid and ethylene signaling pathways, enhancing resistance to pathogens such as *Alternaria brassicicola* and *Botrytis cinerea* by activating the expression of defense-related genes like PR3 and PR4 [10–12]. Many WRKY family transcription factors also participate in plant pathogen defense. For instance, overexpression of rice WRKY67 enhances resistance to bacterial leaf blight [13]. In *Arabidopsis*, WRKY33 is activated by fungal necrotrophs *(A) brassicicola* and *(B) cinerea*, regulating the expression of multiple PR genes and jasmonic acid signaling genes [14]. Conversely, AtWRKY18 and AtWRKY40 negatively regulate *Arabidopsis* powdery mildew caused by *Golovinomyces orontii* [15]. AtWRKY28 and AtWRKY75 are induced by *Sclerotinia sclerotiorum* infection and participate in SA- and JA/ET-dependent defense response pathways [16]. These findings indicate that numerous genes collectively form a complex and sophisticated regulatory network to counter pathogen invasion and infection. The interplay between reactive oxygen species (ROS) and secondary metabolites like lignin represents a central biochemical strategy in plant defense. For instance, H₂O₂ not only acts as a toxic molecule against pathogens but also serves as a signaling intermediate to induce lignin biosynthesis, a process critical for cell wall fortification [17].

Plants can generate reactive oxygen species (ROS) at the corresponding sites to restrict and kill pathogens. This is a rapid response process that minimizes the use of plant resources [18]. ROS are divided into five types, among which H₂O₂ is the most stable and therefore plays a more important role in physiological functions [19]. The production of ROS is mainly mediated by the NADPH oxidase family RBOH. Studies in *Arabidopsis* have found that RbohD is a major participant in ROS production under conditions such as damage, heat, cold or salinity changes [20]. On the one hand, ROS generated by oxidative burst can directly kill pathogens; on the other hand, they act as signaling molecules to interact with Ca²⁺and other factors to induce the expression of a series of plant defense response genes, such as those in the MAPK cascade pathway and PR genes [21]. It is worth noting that high concentrations of ROS can lead to the degradation of plant macromolecules and trigger programmed cell death. During pathogen defense, cell death at the infection site can inhibit further microbial invasion [22]. Therefore, ROS and their synthetic genes play critical roles in plant defense responses.

Another strategy for plant pathogen defense is cell wall thickening, which establishes a physical barrier against pathogen invasion [23]. When pathogen infection occurs, cells perceive signals and induce the expression of lignin synthesis genes, promoting lignin deposition to restrict the invasion of bacteria and toxins. At the same time, this process can also reduce the outflow of cellular substances and weaken bacterial growth [24]. Therefore, it is widely believed that high lignin content has a positive effect on plant disease resistance. For example, after infection by *Erwinia carotovora* subsp. *carotovora*, Chinese cabbage induces the expression of lignin synthesis genes and synthesizes more G and H monolignins [25]. In wheat, the use of rust-derived elicitors can induce the accumulation of syringyl-rich lignin in leaves [26]. The synthesis of lignin and related compounds, as well as the expression of regulatory genes, directly determine plant immune responses. For instance, silencing the expression of *Gh4CL30* promotes the accumulation of caffeic acid, ferulic acid, G and S lignin monomers, enhancing cotton resistance to *Verticillium wilt* [27]. The lignin precursor component 4-hydroxycinnamic acid synthesis gene *OsPAL02* functions in inhibiting *Xanthomonas* and promoting the enrichment of beneficial *Pseudomonas* [28]. Additionally, anionic peroxidases (such as *prxA3a*) are candidate factors for lignin-forming peroxidases. In the presence of H₂O₂, peroxidases catalyze the formation of monolignol radicals, which subsequently disperse to the cell wall and couple to form lignin. Therefore, lignin-forming anionic peroxidases may also have potential functions in plant disease resistance [29].

In this study, we analyzed the resistance response genes of *P. sibirica* L. through RNA-seq of pathogen-infected leaves, heterologously overexpressed the pathogen-induced gene *PsWRKY75* in poplar to analyze its disease resistance function, and finally found that PsWRKY75 can enhance plant disease resistance by directly regulating the expression of the ROS-producing gene *PsRbohB* and the lignin-forming anionic peroxidase *PsLFP1*.

## Results

### Pathogen infection induces the expression of a large number of disease-resistant genes in *P. sibirica* L.

**Fig. 1:**
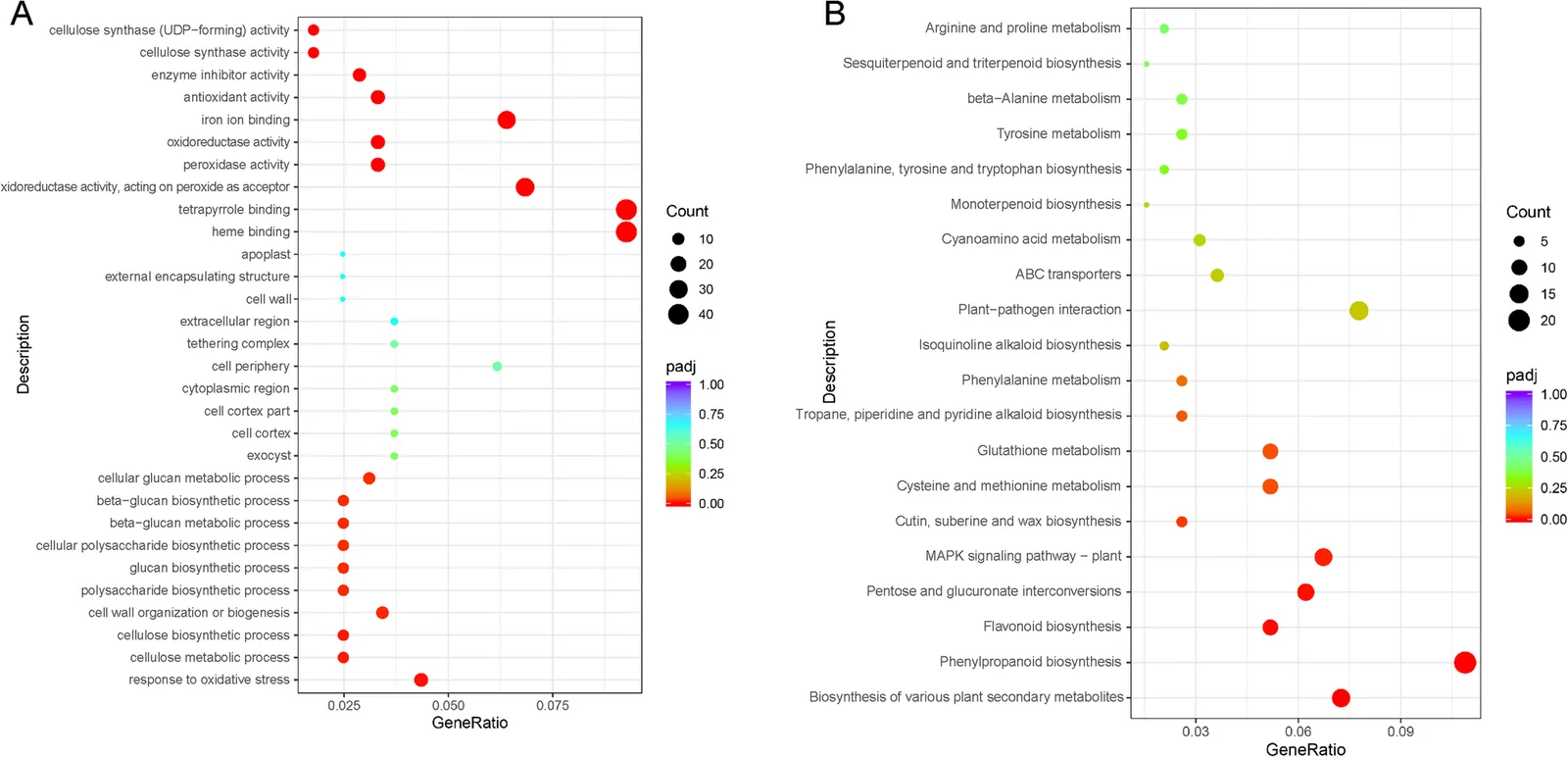
KEGG and GO enrichment analyses of RNA-seq data. **A** KEGG bubble plot; (**B**) GO enrichment analysis bubble plot

**Fig. 2:**
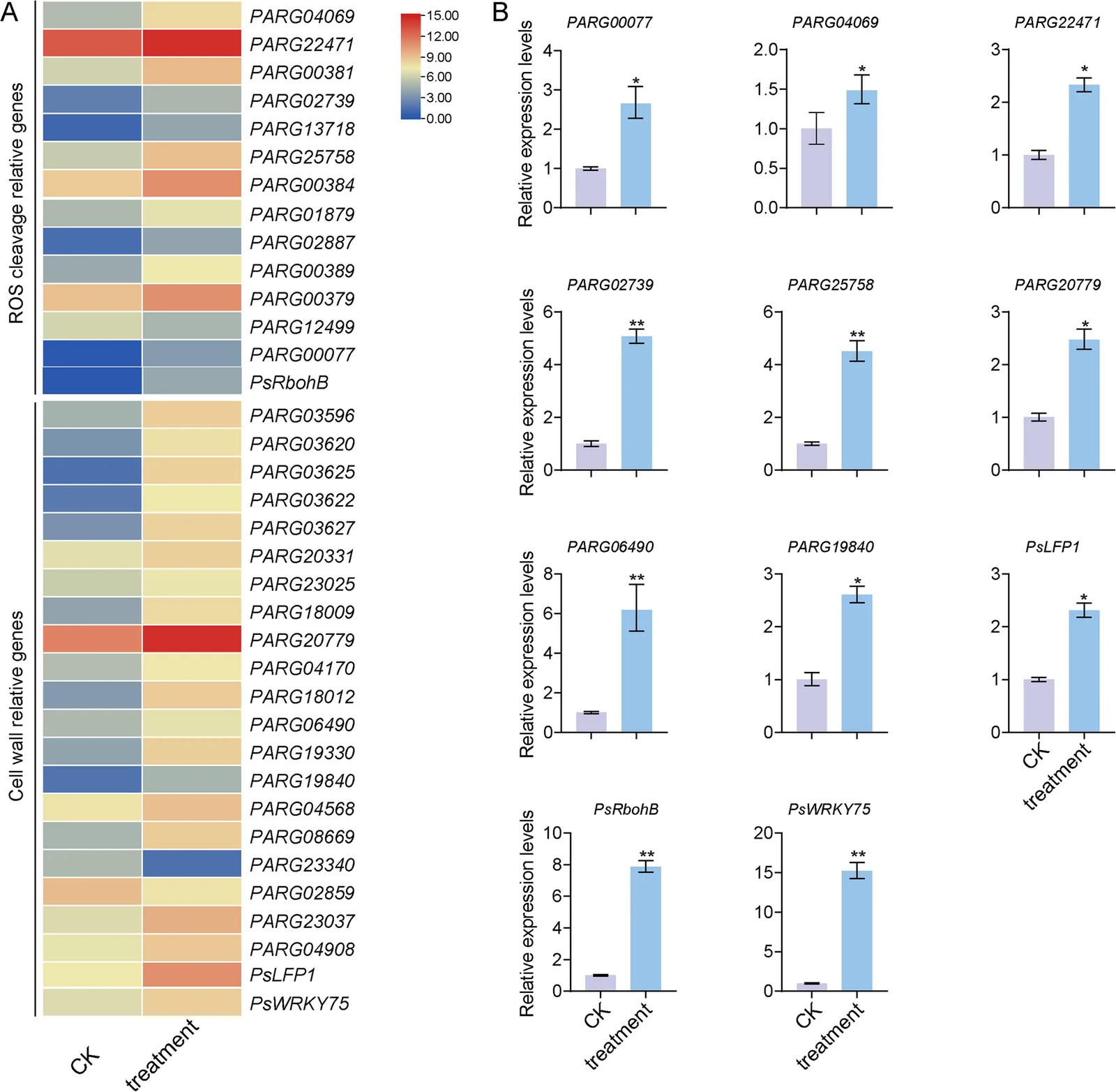
Heatmap and qRT-PCR analysis of ROS scavenging and cell wall synthesis-related genes in DEGs. **A** Heatmap of ROS scavenging and cell wall-related genes; (**B**) qRT-PCR analysis of partial DEGs. Student’s *t*-test was used for significance analysis, * indicates *p* < 0.05 and ** indicates *p* < 0.01

In order to analyze the changes in gene expression of *P. sibirica* L. after pathogen infection, we inoculated and cultured the leaves of healthy *P. sibirica* L. with the canker pathogen *Dothiorella gregaria* Sacc., which establishes a compatible interaction with both *P. sibirica* and poplar, and conducted RNA-seq analysis on the leaves in the inoculated area. Compared with the control, 890 differentially expressed genes (DEGs) were identified in the pathogen-treated group, with 700 upregulated and 190 downregulated (Table S2). KEGG enrichment analysis further highlighted core biological pathways underlying *P. sibirica* L.’s response to pathogenic invasion. Figure 1A shows the top 20 significantly enriched pathways, and these pathways indicate that many DEGs act on lignin synthesis, the MAPK signaling pathway, and plant-pathogen interaction. Further GO annotation and enrichment analysis of DEGs showed that many terms were related to cell wall synthesis and metabolism, such as Cellulose synthase activity, Cell wall, Cellular glucan metabolic process, Cell wall organization or biogenesis; in addition, some terms were related to the ROS scavenging process, such as Antioxidant activity, Peroxidase activity, Response to oxidative stress (Fig. 1B). We selected some ROS scavenging- and cell wall biosynthesis-related genes for heatmap analysis, and the results showed that the expression levels of most genes were up-regulated after pathogen invasion (Fig. 2A), indicating that these genes played a positive role in the disease resistance process of *P. sibirica* L. leaves. Further, we selected some of these genes for RT-qPCR detection, and the results were consistent with the conclusions of the RNA-seq analysis (Fig. 2B).

### *PsWRKY75* is a potential regulatory gene for disease resistance in *P. sibirica* L.

**Fig. 3:**
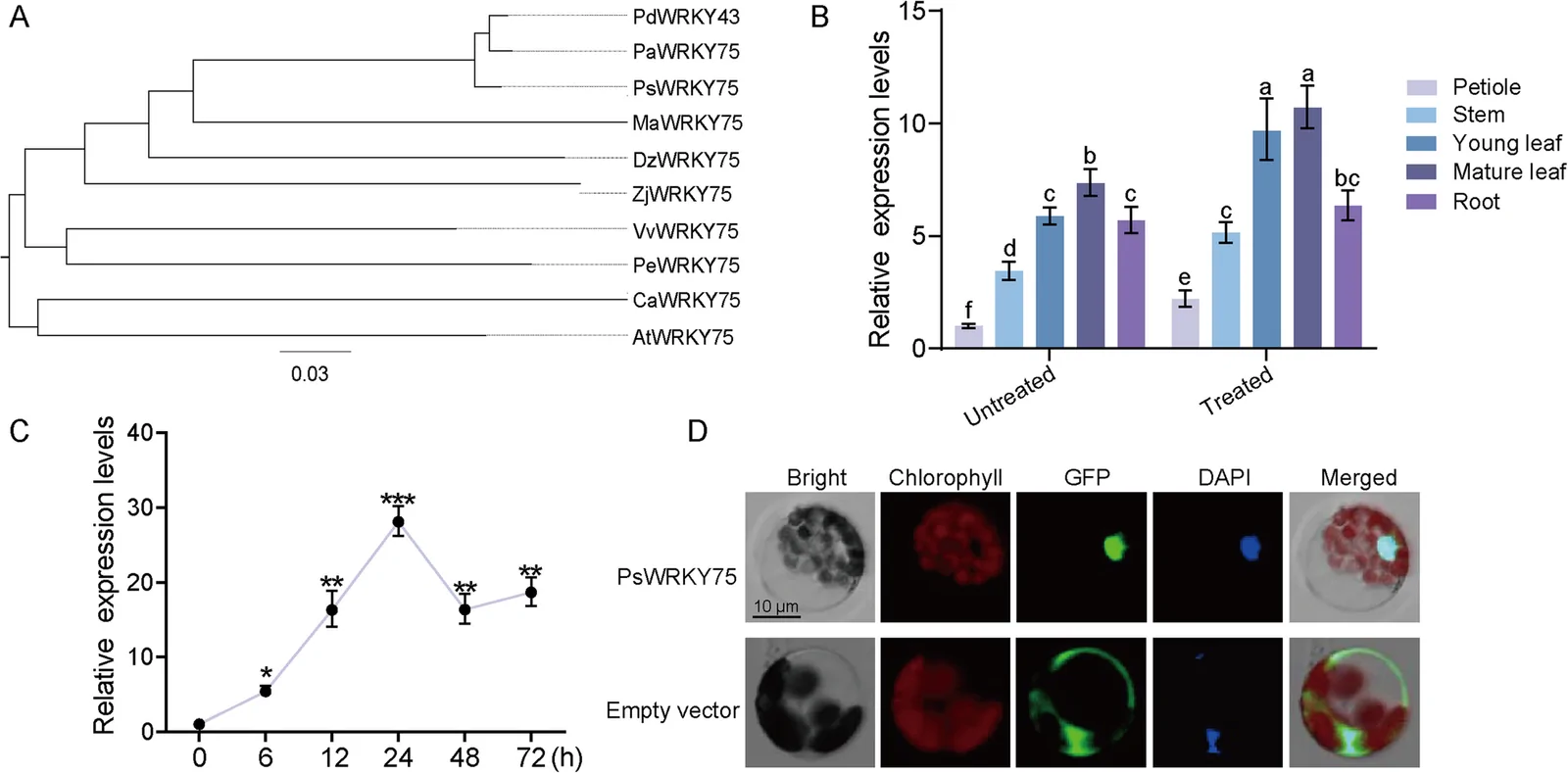
Biological characterization analysis of *PsWRKY75*. **A** Phylogenetic analysis of *PsWRKY75*, with WRKY genes from *Prunus dulcis*, *Prunus avium*, *P. sibirica* L., *Malus sylvestris*, *Durio zibethinus*, *Ziziphus jujuba*, *Vitis vinifera*, *Populus euphratica*, *Corylus avellana*, and *Arabidopsis thaliana* listed from top to bottom; (**B**) Tissue expression pattern of *PsWRKY75* analyzed by qRT-PCR; (**C**) Time-course expression profiles of *PsWRKY75* in leaves after pathogen inoculation at 0, 6, 12, 24, 48 and 72 h; (**D**) Subcellular localization of *PsWRKY75*

Among the DEGs in RNA-seq analysis, we found that the expression level of *PsWRKY75* was significantly up-regulated after treatment (Fig. 2A). Previous reports have shown that *WRKY75* plays a crucial role in pathogen defense in species such as tomato, *Arabidopsis*, and cassava [30, 31]. Therefore, we speculated whether *PsWRKY75* also mediates disease resistance in *P. sibirica* L. The results of RT-qPCR showed that the expression level of *PsWRKY75* was significantly up-regulated in the leaves after pathogen treatment (Fig. 2B). The phylogenetic analysis of *PsWRKY75* showed that it has the closest relationship with PaWRKY75 and PdWRKY43 from *Prunus avium* and *Prunus dulcis*, respectively, suggesting that they have similar biological functions (Fig. 3A).

To further characterize the spatiotemporal expression pattern of *PsWRKY75*, we analyzed its transcript levels in different tissues (leaves, stems, roots, petioles) and at different time points (0, 6, 12, 24, 48, 72 h) after pathogen inoculation using qRT-PCR. Tissue expression pattern of *PsWRKY75* indicated that it was mainly highly expressed in root, young leaves, and old leaves, followed by a certain abundance in stems, and the lowest expression in petioles (Fig. 3B). Time-course analysis revealed that *PsWRKY75* was rapidly induced by pathogen infection: its expression began to increase significantly at 6 h post inoculation, peaked at 24 h, and remained at high levels until 72 h (Fig. 3C).

The result of subcellular localization showed that *PsWRKY75* was localized in the nucleus, indicating that it functions as a transcription factor to regulate gene expression (Fig. 3D). Taken together, these results suggest that *PsWRKY75* is an inducible disease resistance gene in *P. sibirica* L., as its expression is rapidly and significantly upregulated by pathogen infection.

### *PsWRKY75* enhances plant disease resistance

**Fig. 4:**
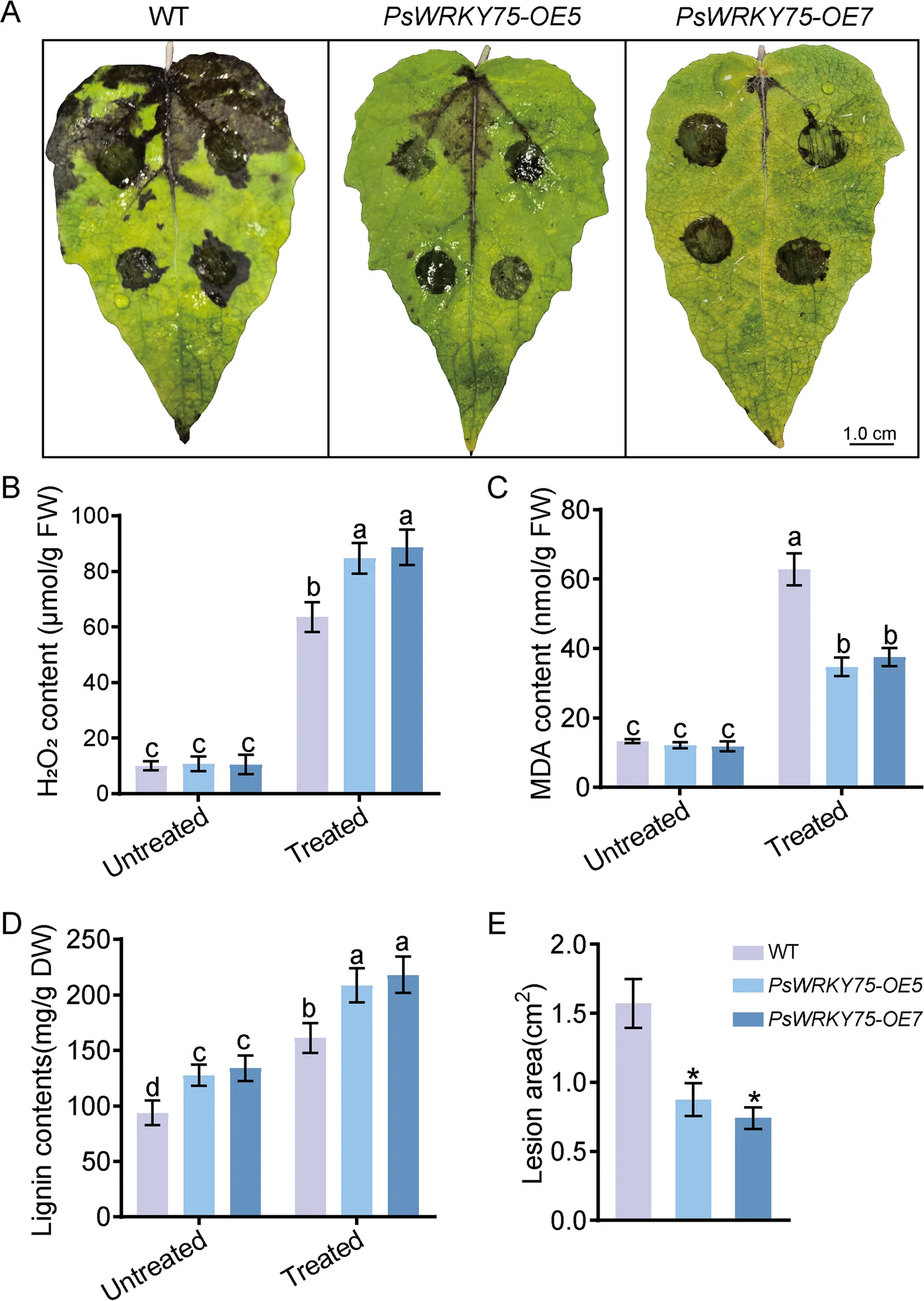
Analysis of disease-resistant phenotypes in *PsWRKY75*-overexpressing poplars. **A** Disease-resistant phenotypes of transgenic poplars 7 days after pathogen treatment; (**B**) H₂O₂ content comparison in poplar leaves under untreated uninfected conditions and pathogen-infected conditions; (**C**) MDA content comparison in poplar leaves under untreated uninfected conditions and pathogen-infected conditions; (**D**) Lignin content comparison in leaf dry weight under untreated uninfected conditions and pathogen-infected conditions; (**E**) Statistical analysis of diseased leaf area. Student’s *t*-test was used for significance analysis, * indicates *p* < 0.05

To further investigate the biological function of *PsWRKY75*, we heterologously overexpressed it in 84 K poplar. RT-qPCR was performed to validate the expression levels in transgenic lines, and two high-expression lines (*PsWRKY75*-*OE5* and *PsWRKY75*-*OE7*) were selected for subsequent analyses (Figure S1). Leaves from these transgenic and wild-type poplars were inoculated with pathogens for 7 days. Results showed that transgenic leaves exhibited significantly enhanced disease resistance compared to WT, with smaller necrotic lesions (Fig. 4A, E).

We measured malondialdehyde content, a marker of membrane lipid peroxidation. To clarify the physiological changes under both normal and pathogen-challenged conditions, MDA content was determined in both untreated and pathogen-infected leaves. Infected WT leaves displayed higher MDA levels than transgenic lines, indicating more severe membrane damage (Fig. 4C). Lower MDA levels in transgenic poplars indicate reduced membrane lipid peroxidation, suggesting that *PsWRKY75* enhances cell membrane stability during pathogen infection—a critical physiological trait for disease resistance.

H_2_O_2_ is produced through oxidative burst to inhibit or kill pathogenic bacteria. H₂O₂ content was measured in both untreated and pathogen-infected leaves to establish baseline levels and induced changes. The determination results of H_2_O_2_ show that *PsWRKY75*-*OE5* and *PsWRKY75*-*OE7* have higher H_2_O_2_ content (Fig. 4B). To further support the increased H₂O₂ accumulation at the molecular level, we analyzed the expression of poplar ROS-related genes, including PagRbohD, PagRbohF, PagCAT1, PagCAT2, PagCAT3, and PagAPX1. qRT-PCR results showed that these genes were significantly upregulated in the overexpression lines, consistent with the elevated H₂O₂ levels (Figure S4).

Additionally, lignin content, a key component for strengthening cell walls against pathogens, increased by approximately 29.4% and 35.3% in the transgenic lines compared to WT (Fig. 4D). To confirm the lignin increase using an independent method, we performed Klason lignin determination on the same samples. The results were consistent with those from the acetyl bromide method, providing complementary evidence for enhanced lignin deposition in the overexpression lines (Figure S3).

**Fig. 5:**
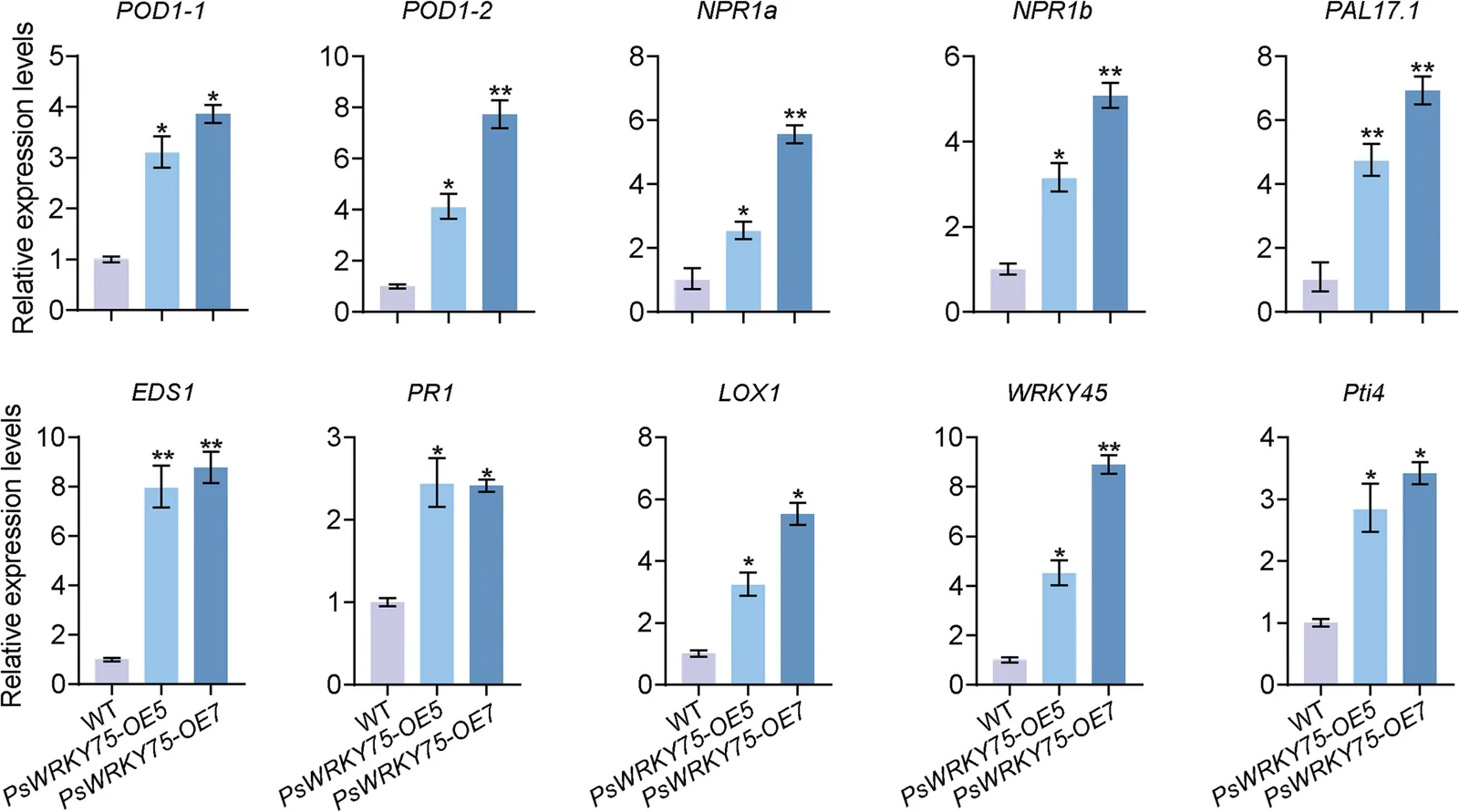
qRT-PCR analysis of disease resistance-related genes in *PsWRKY75* overexpressing poplars. Student’s *t*-test was used for significance analysis, * indicates *p* < 0.05 and ** indicates *p* < 0.01

RT-qPCR analysis revealed that *PsWRKY75* overexpression upregulated several defense-related genes (Fig. 5). For example, *NPR1* is a core regulator of the salicylic acid signaling pathway [32], the expression levels of *NPR1a* and *NPR1b* were significantly upregulated in the poplars overexpressing *PsWRKY75*. *PAL17.1*, a member of the phenylalanine ammonia-lyase family that controls lignin synthesis, was also induced in the transgenic poplars [33]. In addition, genes related to plant disease resistance such as Enhanced disease susceptibility 1 (EDS1), Pathogenesis-related protein 1 (PR1), and Lipoxygenase1 (LOX1) were significantly induced in the poplars with *PsWRKY75* overexpression [34–36]. The above results indicate that the overexpression of *PsWRKY75* in poplars promotes the H_2_O_2_ and lignin accumulation in plants, thereby enhancing plant disease resistance.

### *PsWRKY75* promotes disease resistance by directly regulating the lignin synthesis gene PsLFP1

**Fig. 6:**
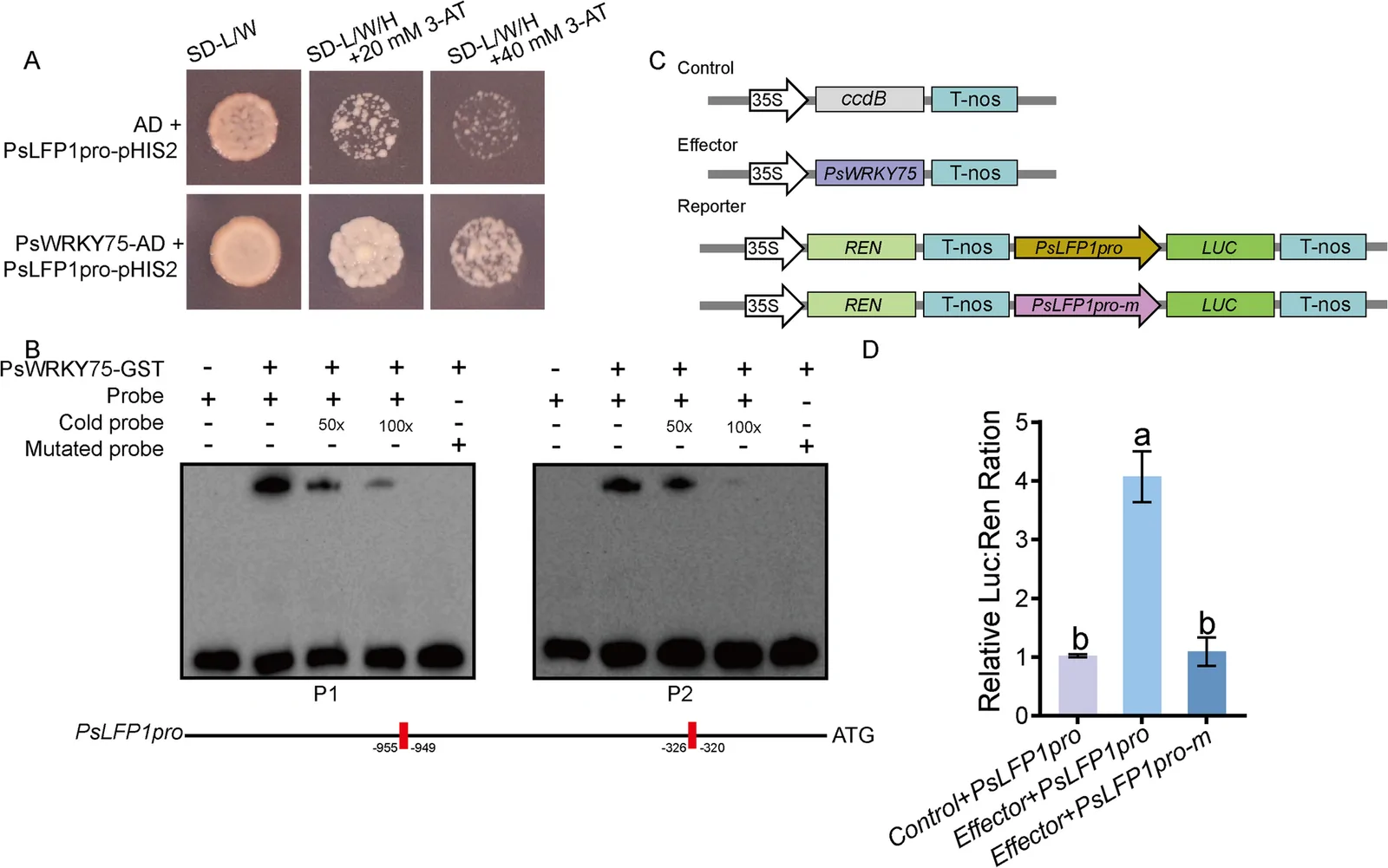
Identification of direct regulation of *PsLFP1* by *PsWRKY75*. **A** Yeast one-hybrid assay; (**B**) EMSA assay to verify the binding of *PsWRKY75* to the W-box in the *PsLFP1* promoter; (**C**) Schematic diagram of effector and reporter in the dual luciferase assay; (**D**) Dual luciferase assay to verify the direct regulation of *PsLFP1* by *PsWRKY75*. Student’s *t*-test was used for significance analysis, * indicates *p* < 0.05 and ** indicates *p* < 0.01

The determination of lignin content showed that the leaves of *PsWRKY75*-*OE* poplars had significantly higher lignin content than the WT (Fig. 4D), suggesting that *PsWRKY75* may enhance plant disease resistance by affecting lignin synthesis. We analyzed the promoters of cell wall-related genes in the DEGs of RNA-seq and found that *PsLFP1*, a member of the lignin-forming anionic peroxidase, had upregulated expression after pathogen treatment, and its promoter contained two W-box motifs (5’-TTGACC-3’) specifically bound by WRKY. Analyzing the tissue expression pattern of *PsLFP1* showed that this gene had the highest expression level in leaves, followed by relatively high mRNA abundance in roots. Time-course qRT-PCR revealed that pathogen infection progressively induced *PsLFP1* transcription in leaf tissue: expression significantly increased from 6 h post inoculation, peaked at 48 h, and slightly decreased at 72 h while remaining significantly higher than the 0 h control (Figure S2), which exhibited an obvious temporal lag compared with *PsWRKY75*, implying that *PsWRKY75* functions as an upstream regulator of PsLFP1. These results indicate that *PsLFP1* may be a downstream regulator of *PsWRKY75*. To verify this hypothesis, we conducted interaction analysis using the yeast one-hybrid system, with 20 mM and 40 mM 3-AT added to the selective medium to fully inhibit the growth of negative control yeast. The results showed that *PsWRKY75* could directly bind to the 1500 bp-length promoter of *PsLFP1* in yeast (Fig. 6A). Further, we verified whether *PsWRKY75* could specifically bind to the W-box on the *PsLFP1* promoter through EMSA. The results showed that *PsWRKY75* could bind to both W-box in vitro, and after the two W-box were mutated, the binding ability of *PsWRKY75* to the motifs disappeared, indicating that *PsWRKY75* exerts downstream regulatory functions by binding to the two W-box (Fig. 6B). The dual-luciferase reporter assay in Nicotiana benthamiana confirmed that *PsWRKY75* specifically binds to the *PsLFP1* promoter and activates its expression in planta. Compared with the wild-type promoter, the activation of the mutated *PsLFP1* promoter (with disrupted WRKY binding sites) by *PsWRKY75* was significantly reduced (Fig. 6C and D). These results indicate that *PsWRKY75* directly enhances the expression of the lignin synthesis gene *PsLFP1* to promote plant disease resistance.

To further clarify the regulatory mechanism in poplar, we first identified the poplar ortholog of *PsLFP1*, designated as PagLFP1, via bioinformatics analysis (Figure S5). We further analyzed the expression of the poplar ortholog of *PsLFP1* (PagLFP1) in WT and *PsWRKY75*-overexpressing poplars with or without D. *gregaria* infection. PagLFP1 showed significantly higher basal expression in OE lines compared to WT. Upon pathogen infection, its expression was further upregulated, with a notably higher induction level in OE lines (Figure S4). These data confirm that *PsWRKY75* positively regulates the transcription of its target gene PagLFP1 in poplar under both normal and pathogen-challenged conditions. 

### *PsWRKY75* enhances the disease resistance of *P. sibirica* L. by directly regulating the H₂O₂-producing gene *PsRbohB*

**Fig. 7:**
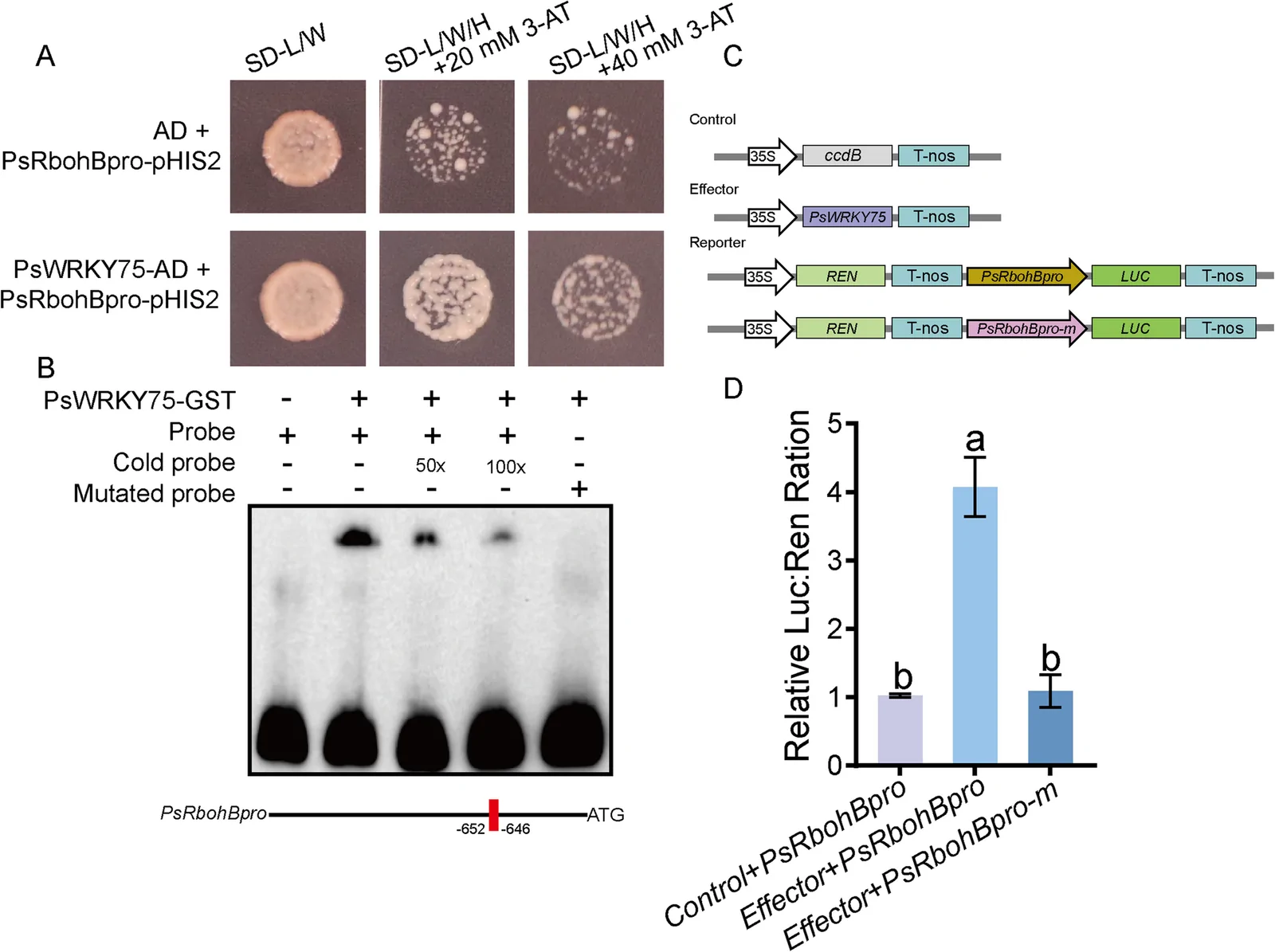
Identification of direct regulation of *PsRbohB* by *PsWRKY75*. **A** Yeast one-hybrid assay; (**B**) EMSA assay to verify the binding of *PsWRKY75* to the W-box in the *PsRbohB* promoter; (**C**) Schematic diagram of effector and reporter in the dual luciferase assay; (**D**) Dual luciferase assay to verify the direct regulation of *PsRbohB* by *PsWRKY75*. Student’s *t*-test was used for significance analysis, * indicates *p* < 0.05 and ** indicates *p* < 0.01

Considering that the *PsWRKY75* transgenic poplar had elevated H₂O₂ levels, we analyzed whether the promoter regions of H₂O₂ generate-related genes in the RNA-seq DEGs contained W-box. A W-box motif (5’-TTGACC-3’) was found at positions − 652 to -646 in the *PsRbohB* promoter (Fig. 7B). Analysis of the tissue expression pattern of *PsRbohB* revealed that it shared a similar expression pattern with *PsWRKY75*, both showing high mRNA abundance in roots and leaves (Figure S2). Consistent with the expression pattern of *PsLFP1*, qRT-PCR time-course assays revealed that pathogen infection persistently induced the transcription of *PsRbohB* in leaf tissue. Its transcript abundance increased markedly starting at 6 h post inoculation and kept rising gradually until peaking at 72 h, which also exhibited obvious temporal lag compared with *PsWRKY75*, suggesting that *PsRbohB* is another putative downstream target of *PsWRKY75* (Figure S2). Additionally, *PsRbohB* was upregulated in infected leaves of *P. sibirica* L. (Fig. 2B). Therefore, we hypothesized that *PsWRKY75* might directly regulate PsRbohB. To test this hypothesis, we performed a Y1H assay by transforming the *PsRbohB* promoter into yeast strain AH109. Yeast cells co-transformed with *PsWRKY75* and the *PsRbohB* promoter grew normally on selective media supplemented with both 20 mM and 40 mM 3-AT, while negative control yeasts showed no growth at these concentrations. These results confirm that *PsWRKY75* directly binds to the *PsRbohB* promoter (Fig. 7A). EMSA confirmed that the *PsWRKY75*-GST fusion protein specifically binds to the W-box motif in the *PsRbohB* promoter but not to mutant. After adding cold probes at different concentrations, the binding of the protein to the probe competitively decreased (Fig. 7B). Finally, we confirmed the direct positive regulation of *PsRbohB* by *PsWRKY75* in planta using a dual-luciferase reporter assay in Nicotiana benthamiana. Compared to the wild-type *PsRbohB* promoter, the activation of the mutated promoter (with disrupted WRKY binding sites) by *PsWRKY75* was significantly abolished. These results demonstrate that *PsWRKY75* specifically binds to the WRKY cis-elements in the *PsRbohB* promoter to activate its expression (Fig. 7C and D). The above results indicate that *PsWRKY75* directly and positively regulates the expression of *PsRbohB*, enhancing H₂O₂ accumulation in plants, thereby improving the disease resistance of *Prunus sibirica* L.

To further verify the regulatory effect of *PsWRKY75* in poplar, we identified the poplar ortholog of *PsRbohB*, namely *PagRbohB*, via bioinformatics analysis (Figure S5). We analyzed the expression of PagRbohB in WT and *PsWRKY75*-overexpressing poplars with or without D. *gregaria* infection. PagRbohB showed significantly higher basal expression in OE lines compared with WT under normal conditions. Upon pathogen infection, their expression levels were further significantly upregulated, and the induction amplitudes were distinctly higher in *PsWRKY75*-overexpressing lines (Figure S4). These data confirm that *PsWRKY75* positively regulates the transcription of PagRbohB in poplar under both normal and pathogen-stressed conditions.

## Discussion

Disease threat is one of the major biotic stresses faced by plants, which seriously affects yield and quality in agricultural and forestry production [37]. *P. sibirica* L. is a unique nectariferous, medicinal, and ornamental plant in Asia, but its growth in nature is severely affected by diseases [38]. In this study, transcriptomics combined with molecular biological approaches was applied to dissect the dynamic gene expression changes in *P. sibirica* leaves during *D. gregaria* infection, aiming to elucidate the regulatory mechanism of the core disease-resistant transcription factor *PsWRKY75*. Spatiotemporal expression analysis revealed that *PsWRKY75* exhibited distinct tissue-specific and temporal expression patterns under pathogen stress, with significant induction at the early and middle stages of infection. It was found that *PsWRKY75* can enhance plant disease resistance by positively regulating the expression of the NADPH oxidase gene *PsRbohB* and the lignin biosynthesis related gene *PsLFP1*.

The members of the WRKY family are widely involved in multiple biological regulatory processes, including plant pathogen defense [39]. WRKY transcription factors are well-documented core regulators of plant immune responses and participate in diverse biotic stress signaling pathways. For example, *Arabidopsis* AtWRKY18 and AtWRKY40 can defend against powdery mildew caused by *Golovinomyces orontii* [15]. AtWRKY4 enhances resistance to necrotrophic and biotrophic pathogens, while AtWRKY3 enhances resistance to the necrotrophic fungal pathogen *B. cinerea*, and pathogen-induced PR1 is suppressed by overexpression of AtWRKY3 and AtWRKY4 [40]. WRKY75 orthologs across species contribute to disease resistance through conserved mechanisms. In Brassica napus, BnaWRKY75 overexpression strengthens resistance to Sclerotinia sclerotiorum, whereas its silencing impairs defense [41]. Similarly, Arabidopsis AtWRKY75 confers resistance to *B. cinerea* and *A. brassicicola* by modulating jasmonic acid pathway genes [31]. The orthologous gene FaWRKY1 of *Arabidopsis* WRKY75 in Fragaria×ananassa is associated with oxidative burst and glutathione-S-transferase (GST) induction, thus acting as a positive regulator of defense against *Colletotrichum acutatum* infection [42]; in kiwifruit, virus-induced gene silencing of AcWRKY75 moderately enhances resistance to *P. syringae* pv. *Actinidiae* [43]. These interspecific functional differences imply that WRKY75 family members exhibit species-specific regulatory diversity, and the immune mechanism of woody plant WRKY75 remains largely unclear. Our present findings identify the positive regulatory role of WRKY75 in fungal disease resistance in *P*. *sibirica*, enriching the functional understanding of woody WRKY75 genes.

Given that the stable genetic transformation system of *P. sibirica* has not been well established and suffers from low transformation efficiency, we adopted poplar as a heterologous expression system. As a classic woody model plant, poplar has a mature genetic transformation system and a close genetic relationship with *P*. *sibirica*, which guarantees the reliability of heterologous functional verification. In this study, both unstressed control and pathogen-infected treatments were set in transgenic poplar materials. In addition, we will further optimize the genetic transformation system of *P. sibirica* in subsequent research to verify the biological function of *PsWRKY75* in its native host. Our results show that overexpression of *PsWRKY75* in poplar enhances plant resistance to pathogen (Fig. 4), accompanied by improved ROS accumulation and increased lignin deposition under pathogen stress, and *PsWRKY75* directly regulates the NADPH oxidase gene *PsRbohB* and lignin biosynthesis relative gene *PsLFP1*. In future work, we will continue to optimize the genetic transformation system to verify the native biological function of *PsWRKY75* in its original host.

Plant early-stage pathogen defense involves the production of reactive oxygen species (ROS), including peroxides and superoxides [44]. Current research indicates that ROS generated by oxidative burst primarily originate from plasma membrane NADPH oxidase and cell wall peroxidases [17, 45]. NADPH oxidase, also known as respiratory burst oxidase, responds to various biotic, abiotic stresses, and developmental processes [46]. For instance, Arabidopsis AtrbohD and AtrbohF are crucial for the oxidative burst that takes place when avirulent strains—specifically the bacterial and oomycete pathogens *P. syringae* and *Hyaloperonospora parasitica*—are encountered [47]. Time-course expression analysis showed that *PsRbohB* was significantly induced at 6–48 h after *D*. *gregaria* infection (Fig. 2). Additionally, *PsWRKY75*-overexpressing exhibited significantly higher H₂O₂ accumulation than WT plants under pathogen infection, while no obvious differences were observed under normal growth conditions (Fig. 4), suggesting that *PsWRKY75* enhances plant disease resistance by regulating *PsRbohB* to generate pathogen-induced ROS, rather than causing constitutive physiological alteration. Given that ROS act as signaling molecules in multiple plant biological processes, it is reasonable to infer that increased *PsRbohB* expression may further influence salicylic acid (SA) levels to mediate disease resistance. Studies have shown that H₂O₂ produced by NADPH oxidase is one of the signaling pathways for SA synthesis [48].

In plant disease resistance mechanisms, the synthesis and accumulation of lignin provide a physical barrier against pathogens [49, 50]. Consequently, plant lignin biosynthesis genes and lignin levels increase after pathogen infection [51]. In *Arabidopsis*, knockout of lignin synthesis genes such as PAL, COMT, and CAD moderately weakens disease resistance against hemibiotrophic bacterial pathogen *P. syringae*, necrotrophic fungal pathogen *A. brassicicola*, and biotrophic fungal pathogen *Blumeria graminis* [52–54]. Our study shows that the lignin-forming anionic peroxidase gene *PsLFP1* is directly positively regulated by *PsWRKY75* and its expression significantly upregulates after pathogen infection in *P. sibirica* L. (Figures 2 and 6). Tissue-specific detection confirmed that *PsLFP1* was predominantly expressed in leaf tissues. Previous research suggests that lignin-related peroxidases limit pathogen invasion of host tissues by catalyzing cross-linking of cell wall components, forming physical barriers in response to stimuli such as wounding and pathogen interactions. This process is mediated by lignin-forming anionic peroxidase-dependent H₂O₂ cross-linking of cell wall components [4]. Lignin content measurements in *PsWRKY75*-overexpressing poplar also show higher levels compared to WT (Fig. 4E). The upregulation of *PsWRKY75* leads to H₂O₂ accumulation, which not only directly inhibits pathogen growth but also serves as a signaling molecule to activate *PsLFP1* expression. This cascade highlights a physiological feedback loop where H₂O₂ triggers lignin deposition, while lignin provides a physical barrier to restrict pathogen invasion. Therefore, enhancing cell wall cross-linking and increasing tissue lignin content are critical strategies for *P. sibirica* L. to resist pathogen invasion.

**Fig. 8:**
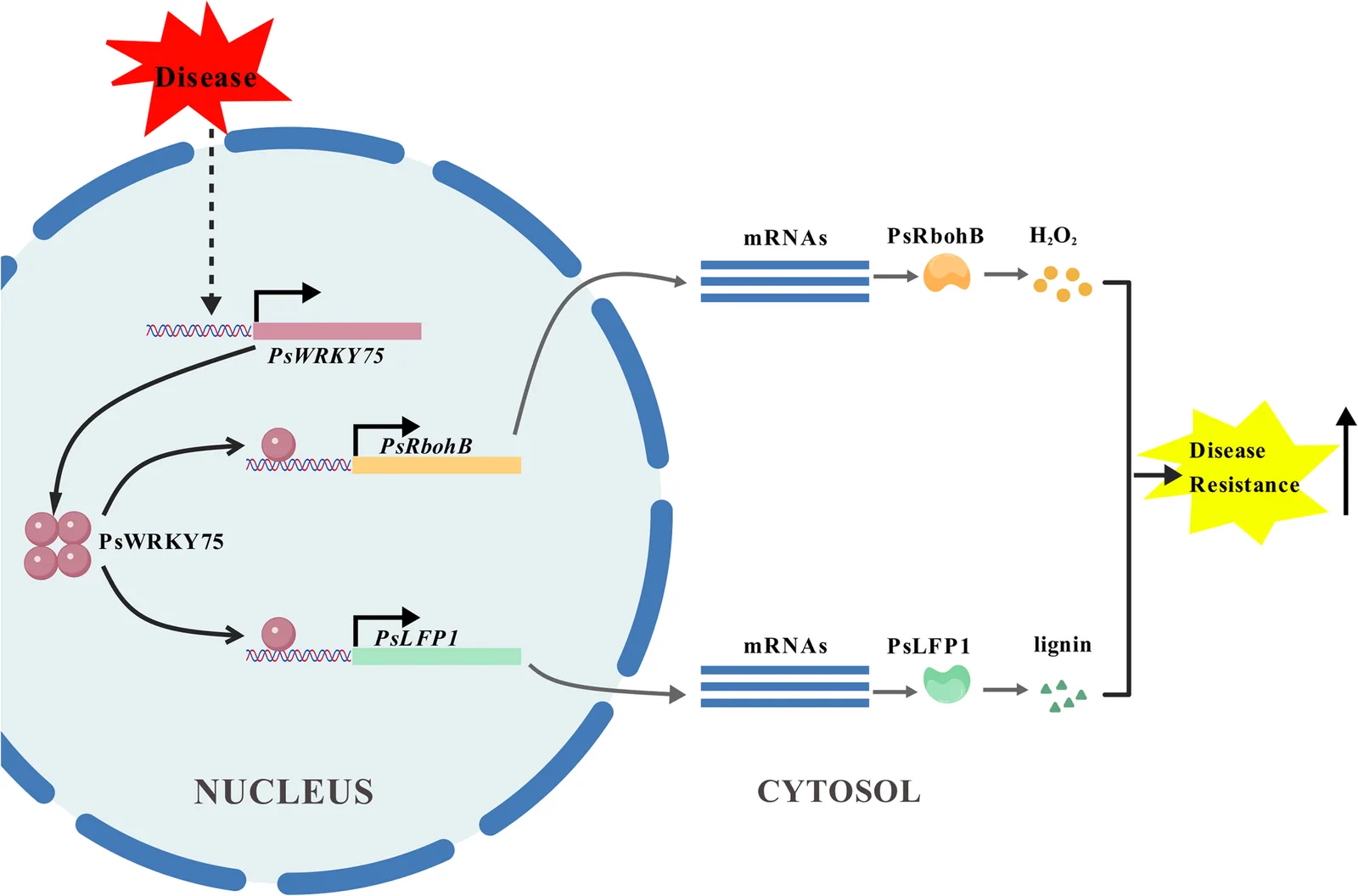
Model of the *PsWRKY75*-mediated disease resistance pathway in *P*. *sibirica* L. Pathogen invasion induces upregulation of *PsWRKY75*, a transcription factor that directly binds to the promoter W-box elements of the H₂O₂-generating gene *PsRbohB* and lignin biosynthesis gene *PsLFP1*, activating their expression. PsRbohB promotes H₂O₂ accumulation to directly inhibit pathogens and activate defense signaling, while PsLFP1 induces lignin deposition to strengthen the cell wall physical barrier. Solid arrows indicate direct regulatory or promotional relationships verified by experiments, while dashed arrows are not involved in this study

In summary, we propose a pathogen resistance pathway in *P. sibirica* L.: upon pathogen invasion, increased *PsWRKY75* expression enhances positive regulation of the ROS generating gene *PsRbohB* and lignin-forming gene *PsLFP1*, leading to stress-dependent elevated ROS levels, strengthened cell wall cross-linking, and increased lignin content, ultimately enhancing resistance to natural pathogen infections. This is shown in Fig. 8, created with https://BioGDP.com [55]. Collectively, our study uncovers a transcription factor-mediated physiological mechanism integrating biochemical pathways of ROS and lignin, offering new perspectives on plant disease resistance at the interface of gene regulation and metabolic adaptation. Our findings reveal a novel transcription factor-mediated ROS-lignin synergistic defense mechanism in woody plants, providing valuable genetic resources and a theoretical basis for disease resistance improvement of forest trees.

## Materials and methods

### Plant materials and growth conditions

The seedlings of *P. sibirica* L., donated by Ningxia Tiancheng Funong Chinese Herbal Medicine Technology Development Co., Ltd., were cultured in a greenhouse under a 16 h light/8 h dark cycle at a light intensity of 150 µmol·m⁻²·s⁻¹ and a constant temperature of 24 °C. The aseptic seedlings of 84 K (*Populus alba* × *P. glandulosa* clone 84 K) poplar were propagated using the woody plant medium (Hopebio, Qindao, China), and cultured in an incubator at a light intensity of 100 µmol·m⁻²·s⁻¹ and a constant temperature of 25℃. For the disease susceptibility experiment, plant leaves with consistent growth status were selected, and a hole puncher was used to gently press on the leaves to create wounds. After inoculating an equal amount of pathogen liquid on the wounds, the leaves were placed on filter paper in a water-saturated state and cultured at 28℃. Three days after inoculation, the RNA of the leaves of *P. sibirica* L. was extracted for RNA-seq, and a liquid without pathogen was set as the control. In the disease susceptibility experiment of transgenic poplar, the infection area was observed 7 days after inoculation, and the physiological data were measured.

### Pathogen culture, inoculation assays, and tissue sampling

The pathogen used in this study, *Dothiorella gregaria* Sacc., is a broad-host-range canker pathogen that forms a compatible pathogenic interaction with both *P. sibirica* and poplar [56]. The fungus was routinely cultured on potato dextrose agar (PDA) medium at 25℃ for 7 days for subsequent inoculation [56].

For *P*. *sibirica* inoculation, healthy leaves from 3-month-old seedlings were selected for treatment. Mycelial plugs (6 mm in diameter) were placed on the surface of excised leaves, with three leaves assessed per plant. All leaves were incubated in Petri dishes lined with moist filter paper in a humid chamber for 3 days [57, 58]. The lesion area and total leaf area were measured and calculated using Adobe Photoshop software.

For RNA-seq and qRT-PCR sampling of *P*. *sibirica*: To comprehensively characterize the spatiotemporal expression pattern of disease-resistant genes, two sets of tissue samples were collected in this study. For tissue-specific expression analysis, different tissues of healthy 3-month-old *P*. *sibirica* seedlings were harvested, including petiole, stem, young leaf tissues around the inoculated region, mature leaf tissues around the inoculated region, and root. For time-course expression analysis, leaf tissues around the inoculated region were collected at a series of time points: 0, 6, 12, 24, 48, and 72 h post-inoculation. Leaves treated with sterile water at the corresponding time points were set as mock controls. All collected samples were immediately frozen in liquid nitrogen and stored at − 80℃ for subsequent total RNA extraction and qRT-PCR detection.

For poplar pathogen inoculation, leaves from 3-month-old wild-type (WT) and *PsWRKY75*-overexpressing poplar plants were inoculated with *D*. *gregaria* using the identical inoculation procedure described above.

For qRT-PCR sampling of transgenic poplar, leaf tissues from WT and *PsWRKY75*-OE lines were collected with or without *D*. *gregaria* inoculation to assess gene expression under both normal and pathogen-stressed conditions. Samples were rapidly frozen in liquid nitrogen and stored at − 80℃ prior to RNA isolation. These samples were used for detecting the transcript levels of *PsWRKY75* and poplar endogenous defense-related genes, including key representative *PagLFP1*, *PagRbohB*, *PagRbohD*, *PagRbohF*.

All experiments were performed with at least three biological replicates. Significant differences were determined using Student’s t-test at *P* ≤ 0.05 via Origin 8.0 software (v8.0725, OriginLab Corp., Northhampton, MA, USA).

### Extraction of genomic DNA (gDNA) and total RNA, and real time-quantitative PCR (RT-qPCR)

For promoter cloning and transgenic poplar validation, genomic DNA (gDNA) was isolated via the CTAB protocol [59]. Plant tissues were ground in 2× CTAB buffer, followed by chloroform-mediated separation of proteins and polysaccharides, and DNA precipitation with isopropanol. Finally, the DNA was dissolved in ddH₂O.

Total RNA was extracted using the Plant Total RNA Extraction Kit (Bioflux, Beijing, China), and cDNA was obtained by reverse transcription using the Evo M-MLV RT Mix Kit (Accurate Biology, Hunan, China). On this basis, Real Time PCR East™-SYBR Green II (Foregene, Chengdu, China) was used for the RT-qPCR experiment, and the expression levels were calculated using the 2^ΔΔCt^ method [60]. All gene-specific primers are shown in Table S1.

### Gene, promoter cloning and vector construction

The coding sequence of *PsWRKY75* and the 1500 bp promoters of *PsRbohB* and *PsLFP1* were cloned using *ApexHF* HS DNA Polymerase CL (Accurate Biology, Hunan, China). The former used the cDNA of *P. sibirica* L. as a template, while the latter two used gDNA as templates. The PCR procedure was as follows: pre-denaturation at 94 °C for 5 min, followed by 34 cycles of 95 °C for 15 s, 56 °C for 15 s, and 72 °C for 1 min, with a final extension at 72 °C for 5 min. Subsequently, *PsWRKY75* was cloned into the pK2GW7-3xFLAG vector for overexpression and into pGADT7 for yeast one-hybrid assays. The promoters of *PsRbohB* and *PsLFP1* were cloned into pHIS2 for yeast one-hybrid assays and into pGREENⅡ0800-LUC for dual-Luciferase assays.

### Heterologous overexpression of *PsWRKY75* in poplar and transgenic line identification

The full-length coding sequence of *PsWRKY75* was amplified and cloned into the pK2GW7-3xFLAG overexpression vector under the control of the CaMV 35 S promoter, with kanamycin resistance as the bacterial selection marker and Basta resistance as the plant selection marker. The recombinant vector pK2GW7-3xFLAG-*PsWRKY75* was transformed into *Agrobacterium tumefaciens* strain GV3101 by the freeze-thaw method [61].

Subsequently, *PsWRKY75* overexpressing poplar lines were generated using the Agrobacterium-mediated leaf disc transformation method in *Populus alba* × *P*. *glandulosa* clone 84 K, following a previously described protocol with minor modifications [62]. Briefly, sterile leaf discs were co-cultivated with transformed *Agrobacterium* in the dark, then transferred to selection medium containing 3 mg/L Basta for transformant screening. Surviving shoots were rooted and acclimatized in soil.

Total RNA was extracted from leaves of putative transgenic and wild-type (WT) poplar plants, and the expression level of *PsWRKY75* was determined by qRT-PCR using poplar *UBQ* as the internal reference gene. Two independent T0 transgenic lines with the highest *PsWRKY75* expression levels, designated OE5 and OE7, were selected for subsequent functional analyses.

### RNA-seq

Novogene (Beijing, China) constructed the cDNA library for the samples and sequenced using the Illumina HiSeq X Ten platform. The obtained raw data were evaluated for quality by FastQC. The transcripts per million (TPM) were evaluated by Stringtie [63]. Subsequently, EdgeR [64] was used to analyze the differentially expressed genes (DEGs) between the treatment and the control, and Tbtools Ⅱ was used for GO and KEGG analyses [65]. All the RNA-seq data have been uploaded to the National Genomics Data Center (NGDC; https://bigd.big.ac.cn/bioproject). The BioProject accession numbers are as follows PRJCA040551.

### Dual-luciferase assay

The dual-luciferase assay was performed using the Luciferase Reporter Assay Kit (Biovision, San Francisco, CA, USA). The promoters of *PsRbohB* and *PsLFP1* (wild-type and mutated versions with disrupted WRKY binding sites) were cloned into the pGREENⅡ0800-LUC vector as reporters. The overexpression vector of *PsWRKY75* was used as the effector. Both reporter and effector constructs were transformed into *Agrobacterium tumefaciens* strain GV3101. The transformed strains were cultured in YEP liquid medium until the OD₆₀₀ reached approximately 0.8. The *Agrobacterium* cells were collected by centrifugation, resuspended in infection buffer (10 mM MgCl₂, 10 mM MES, 100 µmol/L acetosyringone, pH 5.7), and incubated at 28 °C for 2 h. Equal volumes of reporter and effector suspensions were mixed and co-infiltrated into the leaves of Nicotiana benthamiana. After 2 days of dark treatment followed by 1 day of light incubation, total leaf proteins were extracted. Firefly and Renilla luciferase activities were measured using the Dual-luciferase Reporter System (Synergy H1, BioTek, Winooski, VT, USA).

### Electrophoresis Mobility Shift Assay (EMSA)

The CDS of *PsWRKY75* was constructed into the pGEX-4T-1 vector and transformed into the *Escherichia coli* strain BL21. The purification of the prokaryotic expressed protein was carried out according to the protocol of Harper and Speicher [66]. Briefly, 0.5 mM IPTG was used to induce the protein expression in the transgenic BL21, and the *PsWRKY75*-GST fusion protein was enriched using GSTSep Glutathione Magbeads (YEASEN, Shanghai, China). The probes with a 5’-biotin label and the mutant probes were synthesized by Ykang (Hangzhou, China), and the EMSA experiment was conducted according to the protocol of the LightShift^®^ Chemiluminescent EMSA Kit (Thermo Scientific, Waltham, MA, USA). The sequences of all the probes and primers are shown in Table S1.

### Determination of lignin content

The acetyl bromide method [67] is used for the determination of lignin content. After crushing the dried plant into powder, 10 mg of the sample is taken and added to 2 ml of an acetyl bromide/glacial acetic acid solution (v/v, 1/3). Lignin content was quantified using the acetyl bromide method with modifications for woody tissues [67], and results were normalized to leaf dry weight to reflect physiological accumulation levels. After incubation at 70 °C for 30 min, 0.5 ml of NaOH (0.3 M) is added, and then 0.5 ml of hydroxylamine hydrochloride (0.5 M) is added. After centrifugation at 10,000 g for 10 min, the absorbance is measured at OD_280_. The lignin content is calculated according to the following formula: lignin content = (A280*V)/(20 L▪g^−^¹▪cm^−^¹*W*1000), where V represents the reaction volume and W is the dry weight of the sample.

### Determination of lignin content by Klason method

Klason lignin content was measured according to the standard sulfuric acid hydrolysis method with minor modifications. Briefly, freeze-dried poplar leaf samples were ground into fine powder using a ball mill and passed through a 40-mesh sieve. The powder (100 mg) was transferred to a 15 mL centrifuge tube, and 1 mL of 72% (v/v) H₂SO₄ was added. The mixture was incubated at 30 °C for 1 h with constant shaking, then diluted with 4 mL of distilled water to a final concentration of 1 M H₂SO₄. The tube was autoclaved at 121 °C for 1 h to complete hydrolysis. After cooling to room temperature, the hydrolysate was filtered through a pre-weighed glass fiber filter paper. The residue was washed with hot distilled water until the filtrate was neutral, then dried at 105 °C to constant weight. The Klason lignin content was calculated as the percentage of the weight of the dried residue relative to the initial sample dry weight, expressed as mg/g dry weight (DW) [68].

### Yeast one-hybrid

The 1500-bp promoters of *PsRbohB* and *PsLFP1* were cloned into the pHIS2 vector and co-transformed with *PsWRKY75*-AD into *Saccharomyces cerevisiae* strain AH109 using the lithium acetate method [69]. Transgenic yeast cells were selected on SD-Leu-Trp medium. To optimize the selection conditions and completely inhibit the growth of negative control yeast, a concentration gradient of 3-AT (3-Amino-1,2,4-triazole) was tested, and 20 mM and 40 mM 3-AT were determined as the optimal concentrations for the *PsRbohB* and *PsLFP1* promoter assays, respectively. The binding ability of *PsWRKY75* to the promoters was assessed by growing the yeast on SD-Leu-Trp-His selective medium supplemented with the corresponding optimal concentrations of 3-AT. The empty pGADT7 vector co-transformed with the recombinant pHIS2 vector served as the negative control.

### Measurement of MDA and H_2_O_2_

The MDA content was determined by the thiobarbituric acid (TBA) method [70]. After weighing the leaves and crushing them, 1 ml of 5% trichloroacetic acid was added. Then, the mixture was centrifuged at 4000 g for 10 min, and 200 µl of the supernatant was taken and mixed with 200 µl of 0.67% thiobarbituric acid. After incubation at 100 °C for 30 min, the supernatant was taken, and the absorbance values were measured at OD450, OD532 and OD600. The MDA content was calculated using the following formula: MDA content = [6.45·(A532 − A600) − 0.56*A450]/fresh weight.

The determination of H₂O₂ was performed using the titanium salt colorimetric method [71]. H₂O₂ content was measured using the titanium salt colorimetric method, and data were expressed as µmol/g fresh weight to assess real-time oxidative burst responses. Briefly, plant samples were disrupted in acetone, centrifuged, and the supernatant was collected. Equal volumes of titanium sulfate solution (1.2% Ti(SO₄)₂, w/v) and sulfuric acid solution (1.2 M) were added, mixed thoroughly, and reacted at room temperature for 10 min. The absorbance was measured at OD415, and the H₂O₂ content was calculated using a standard curve.

### Subcellular localization of *PsWRKY75*

In this study, subcellular localization analysis of *PsWRKY75* was performed by dissociating poplar mesophyll cells, which was carried out according to the method of Russell and McCown (1986) [72]. *PsWRKY75* was cloned into a eukaryotic transient expression vector with GFP fluorescence, and the vector was transferred into protoplasts by the PEG method [73]. The subcellular localization of *PsWRKY75*-GFP was observed under a laser confocal microscope(Leica TCS SP5 Ⅱ system).

### Statistical method

The Student’s *t*-test is used for the comparison of differences between groups. Different asterisks represent different significance levels. * indicates *p* < 0.05 and ** indicate *p* < 0.01.

## Supplementary Information

Supplementary Material 1: qRT-PCR analysis of expression levels in *PsWRKY75* overexpression poplars.

Supplementary Material 2: Tissue expression patterns of *PsRbohB* and *PsLFP1*.

Supplementary Material 3: Lignin contents in wild-type (WT) and *PsWRKY75*-overexpressing poplar lines under untreated and D. *gregaria*-infected conditions. Lignin contents were measured in mg/g dry weight (DW). Data represent means ± SD of three biological replicates. Different lowercase letters above the bars indicate significant differences (*p* < 0.05) according to one-way ANOVA followed by Tukey’s multiple comparisons test.

Supplementary Material 4: Sequence alignment and conserved domain analysis of *PsRbohB* and PsLFP1 with their poplar orthologs. A. Multiple sequence alignment of *PsRbohB* with its poplar ortholog PagRbohB. The conserved functional domains, including NADPH_Ox, EF-hand, FAD binding 8, and NAD_binding 6, are marked by red lines above the sequences. Identical and similar residues are shaded in dark blue and light blue, respectively. B.Multiple sequence alignment of PsLFP1 with its poplar ortholog PagLFP1. The conserved secretory peroxidase domain is indicated by a red line above the alignment. Identical and similar residues are shaded in dark blue and light blue, respectively.

Supplementary Material 5: Expression profiles of defense-related genes in wild-type (WT) and *PsWRKY75*-overexpressing poplar lines (*PsWRKY75*-OE5, PagWRKY75-OE7) under untreated and D. gregaria-infected conditions. The relative expression levels of genes involved in lignin biosynthesis (PagLFP1) and ROS metabolism (PagRbohB, PagRbohD, PagRbohF, PagCAT1, PagCAT2, PagCAT3, PagAPX1) were determined by qRT-PCR. Data represent means ± SD of three biological replicates. Different lowercase letters above the bars indicate significant differences (*p* < 0.05) according to one-way ANOVA followed by Tukey’s multiple comparisons test.

Supplementary Material 6: The primer sequences used in this study.

Supplementary Material 7: RNA-seq data of *P. sibirica* L. under pathogen treatment and control groups.

## Data Availability

The data that support the findings of this study are available from the corresponding author upon reasonable request.
